# Mathematical Modeling of Programmatic Requirements for Yaws Eradication

**DOI:** 10.3201/eid2301.160487

**Published:** 2017-01

**Authors:** Michael Marks, Oriol Mitjà, Christopher Fitzpatrick, Kingsley Asiedu, Anthony W. Solomon, David C.W. Mabey, Sebastian Funk

**Affiliations:** Hospital for Tropical Diseases, London, UK (M. Marks, A.W. Solomon, D.C.W. Mabey);; London School of Hygiene and Tropical Medicine, London (M. Marks, A.W. Solomon, D.C.W. Mabey, S. Funk);; ISGlobal, Barcelona, Spain (O. Mitjà);; World Health Organization, Geneva, Switzerland (C. Fitzpatrick, K. Asiedu, A.W. Solomon)

**Keywords:** mass drug administration, MDA, yaws, neglected tropical diseases, NTDs, mathematical modelling, bacterial infection, Treponema pallidum, bacteria, eradication

## Abstract

Yaws is targeted for eradication by 2020. The mainstay of the eradication strategy is mass treatment followed by case finding. Modeling has been used to inform programmatic requirements for other neglected tropical diseases and could provide insights into yaws eradication. We developed a model of yaws transmission varying the coverage and number of rounds of treatment. The estimated number of cases arising from an index case (basic reproduction number [R_0_]) ranged from 1.08 to 3.32. To have 80% probability of achieving eradication, 8 rounds of treatment with 80% coverage were required at low estimates of R_0_ (1.45). This requirement increased to 95% at high estimates of R_0_ (2.47). Extending the treatment interval to 12 months increased requirements at all estimates of R_0_. At high estimates of R_0_ with 12 monthly rounds of treatment, no combination of variables achieved eradication. Models should be used to guide the scale-up of yaws eradication.

Yaws is a bacterial infection caused by *Treponema pallidum* subsp. *pertenue* (*1*). The disease predominantly affects children living in poor, remote communities and results in lesions of the skin, bone, and cartilage. Previously, yaws was widespread throughout the tropics (*2*), but in the 20th century a series of control efforts based on mass treatment and case finding led by the World Health Organization (WHO) is estimated to have reduced the burden of cases worldwide by up to 95% (*3*). Despite these efforts, the disease has resurged in several countries in West and Central Africa, the Pacific, and Southeast Asia.

In 2012, a single dose of azithromycin was shown to be effective treatment for yaws (*4*). The availability of a well-tolerated oral agent has prompted WHO to develop a new eradication strategy, known as the Morges strategy, based on community mass azithromycin treatment (*5*). The strategy is supported by World Health Assembly resolution 66.12, which calls for eradication of yaws by 2020 (*6*). The strategy combines an initial round of total community treatment (TCT) followed by subsequent active case finding and total targeted treatment (TTT) of newly identified patients and their contacts. Pilot studies have shown that community mass treatment with azithromycin is a highly effective strategy for reducing the community prevalence of yaws (*7*,*8*).

Data are limited to inform the optimum coverage and number of TCT or TTT rounds that are required to achieve elimination (i.e., interruption of transmission) of yaws at a local level to facilitate country-level elimination and ultimately global eradication. In India, a national yaws elimination campaign conducted during 1996–2004 resulted in substantial reduction in the prevalence of yaws, sustained interruption of transmission, and nationwide elimination (*9*). This program consisted of case-finding surveys and treatment with parenteral penicillin conducted every 6 months. Although this approach did not include the initial mass treatment round now recommended as part of the Morges strategy, its success indicates that serial rounds of high-coverage treatment might achieve local elimination.

A recent review of important research questions facing the global yaws eradication program has highlighted the need for more accurate data to inform the optimum number and coverage of rounds of TCT and TTT that will be required to achieve yaws eradication (*10*). Mathematical modeling has been used to inform control efforts for several other neglected tropical diseases (*11*–*13*) that are also managed by using community mass treatment strategies, and such approaches could be of value for yaws eradication efforts. In particular, this approach might allow a comparison of the differential effects of alternative mass treatment strategies, which would be difficult to assess by empirical randomized controlled trials because of the size and cost of implementing large-scale cluster randomized studies.

Previous mathematical models for yaws (*14*) have assessed the cost-effectiveness of yaws eradication but have not directly addressed the feasibility of achieving this goal or the number of rounds of treatment that would be required. In this study, we aimed to determine whether the eradication of yaws is feasible based on the Morges strategy and, if it is, the number and coverage of mass treatment rounds needed to achieve the goal.

## Methods

**Figure 1:**
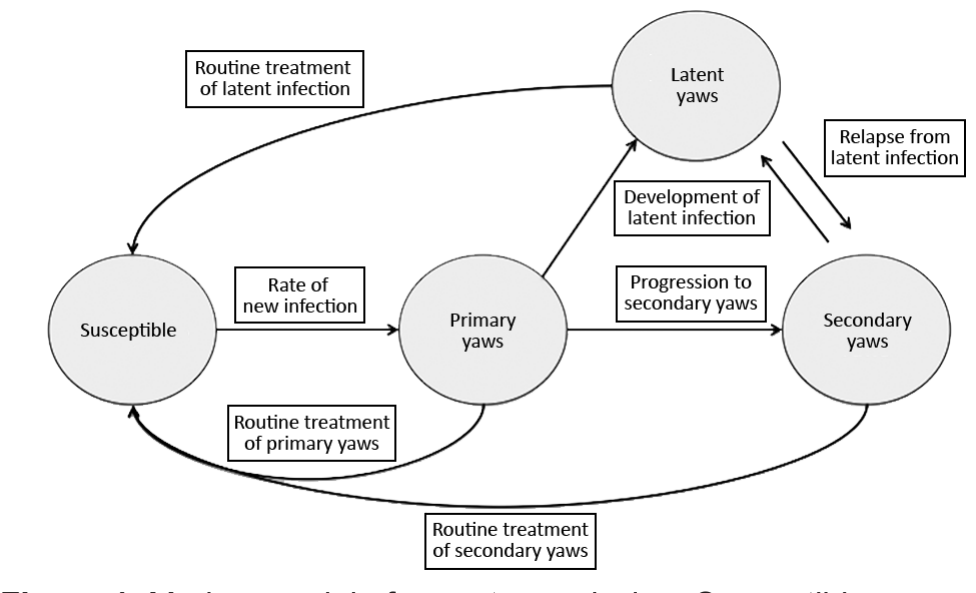
Markov model of yaws transmission. Susceptible persons become infected at a rate dependent on the probability of transmission and the number of persons with infectious primary and secondary yaws. Without treatment, illness progresses from primary disease to either latent yaws or secondary yaws. Persons with latent yaws might relapse to generate secondary cases of infectious yaws in others.

We developed a stochastic Markov model of community-level transmission of yaws (Figure 1). This model treats each stage of the disease as a discrete compartment, with persons moving through each compartment as the disease progresses or is treated. Upon infection, susceptible persons acquire primary disease at a rate that is proportional to the transmission rate and the total number of infectious persons. Persons with primary disease can further transition to secondary disease, at which stage they remain infectious, and both those with primary and secondary disease can transition to latent disease, which is not infectious. Last, those with latent disease can relapse back to secondary infectious disease. The model includes a rate of routine treatment for persons with primary or secondary disease, after which they become susceptible to infection again. The model also includes a lower rate of routine treatment for latent disease, after which the patients also become susceptible to infection again. Unlike previous mathematical models of yaws (*14*), tertiary yaws was not included in the model because such cases are believed not to contribute to transmission (*15*). Because persons might be reinfected many times, we did not consider them to obtain protective immunity after infection or treatment (Technical Appendix).

Although some evidence suggests the existence of a nonhuman primate reservoir for yaws in Africa (*16*), we did not include such infections in our model because there is currently no definitive evidence that the organism responsible for these infections is the same one that causes human yaws or that zoonotic transmission occurs in the real world. We therefore considered the epidemiologic importance of this possible reservoir as minimal when constructing our model.

### Population Size

Estimates of the starting population for each compartment were derived from published population-based yaws prevalence studies (*7*,*17*). We modeled a discrete closed population without addition or reduction through births or deaths.

### Disease Characteristic Variable Estimates

We estimated values for the rates of disease progression between different stages of yaws, including development of and relapse from latent yaws, by using expert opinion, published data, and estimates used in other models (Table 1) (*1*,*14*,*17*). We defined 3 transmission scenarios (low, medium, and high) by using published age-specific treponemal seroprevalence data (*17*), expert opinion, and values used in other yaws models (*14*). Based on these data, we calculated initial estimates of the number of new infections arising from a single index case in a fully susceptible population (the basic reproduction number [R_0_]). Based on the seroprevalence data, we generated R_0_ estimates of 1.25 (low), 1.83 (medium), and 2.4 (high). These estimates were converted to estimates of the probability of transmission after contact between an infectious person and a susceptible persons (β). The mean R_0_ taking account of the full structure of our model, including duration of infection and the size of each starting population, is 1.96, resulting in a mean R_0_ of 1.45 (95% CI 1.01–2.14) for the low transmission settings, 1.95 (95% CI 1.38–2.91) for medium, and 2.47 (95% CI 1.7–3.68) for high transmission. We included a variable to represent the likelihood of a person receiving treatment for yaws in the absence of public health interventions based on published data (*17*).

**Table 1:** Parameters used in modeling treatment coverage required to achieve yaws eradication

Parameter	Parameter estimate	Source of estimate	Comments
Epidemiologic parameters			
R_0_*	1.08–3.32	Derived from published survey data (*17*)	The average number of new cases occurring from a single index case in a fully susceptible population
Monthly probability of progression from primary to secondary disease without treatment	2.78%–5.56%	Derived from expert opinion and previously published models (*1*,*14*)	All untreated persons with primary disease either develop secondary or latent stage disease, and this occurs over a period of 2–6 mo. Untreated persons with latent cases might relapse for a period of >5 y and become actively infectious again.
Monthly probability of progression from infectious to latent disease without treatment	13.9%–27.8%		
Monthly probability of relapse from latent disease to infectious stage without treatment	1%–3%		
Population parameters			
Susceptible at baseline	64%	Derived from published survey data (*17*)	Data derived from multiple pre–mass drug administration surveys conducted in communities where yaws is endemic
Primary yaws at baseline	1.5%		
Secondary yaws at baseline	1.5%		
Latent yaws at baseline	33%		
Mass treatment parameters			
Total community treatment coverage†	65%–95%	Expert opinion and published data on coverage achieved in other mass treatment campaigns (*18*)	Coverage estimates were chosen to reflect the range achieved in real-world mass drug administration programs for other neglected tropical diseases
Total targeted treatment coverage of persons with active cases‡	65%–95%		
Total targeted treatment coverage of persons with latent cases‡	65%–95%		
No. rounds of total community treatment†	1–3		
No. rounds of total targeted treatment‡	0–5		

*R_0_ (basic reproduction number) is the number of new cases arising from a single index case in a fully susceptible population. †Total community treatment consists of mass drug administration to all residents in a community where yaws is endemic regardless of clinical or serologic evidence of disease. ‡Total targeted treatment consists of active case finding and treatment of newly identified persons with yaws and their contacts.

### Mass Drug Administration Variables Estimates

We performed simulation experiments to estimate the impact of a yaws eradication intervention on disease transmission. In line with the Morges strategy (*5*), we considered 2 program components. In the first component, TCT, all persons were considered to have an equal chance of receiving treatment regardless of their infection status. In the second component, TTT, we considered that the coverage achieved among persons with active infection and those with latent infection might differ. Intervention coverage was modeled independently for TCT, with TTT pertaining to persons with active infection and persons with latent infection over a range of plausible estimates (65%–95% population coverage). Mass treatment compliance was simulated as a random, nonsystematic process (i.e., each person had the same chance of receiving treatment, with the likelihood of any 1 person receiving treatment being independent).

We varied the number of treatment rounds of TCT (1–3 rounds) and TTT (0–5 rounds). Where >1 rounds of TTT were implemented, these rounds followed the final round of TCT. In line with the Morges strategy and real-world pilot implementations (*5*,*7*), rounds of mass treatment were spaced at 6-month intervals. We also conducted an analysis with rounds of treatment spaced at 12-month intervals to assess whether annual treatment might also be effective.

We derived estimates of the efficacy of single-dose treatment with azithromycin from randomized controlled trials of azithromycin for the treatment of yaws (Table 1) (*4*). After successful treatment, yaws lesions become noninfectious within 24 hours (*1*); therefore, we considered treatment to be immediately efficacious at the time of mass drug administration, with persons reverting to a susceptible state after treatment.

### Implementing the Model

The model was implemented in R software (*19*). We performed repeated simulations across a range of assumptions about the rate of transmission (equivalent to low, medium, and high estimates of R_0_) and assumptions about mass treatment, varying the coverage and number of mass treatment rounds undertaken (Table 1).

For each combination of disease and intervention parameters, we performed 1,000 simulation experiments. Within each combination of transmission and treatment assumptions, we varied other disease-specific variables (e.g., rate of progression and relapse and treatment in the absence of intervention) across the range of parameter estimates. The model was run for an initial period of 50 months to achieve a steady state with yaws eradication interventions modeled to commence after this initial period. The model then ran for a further 100 months (online Technical Appendix).

### Assessing Outcomes

For each run of the model, we recorded whether eradication was achieved. Eradication was defined as no cases of infectious or latent yaws at the end of the model run. The eradication probability was defined as the percentage of runs within each permutation of model characteristics where eradication was achieved. All analyses were performed by using R version 3.2.2.

## Results

The model generated a total of 6,174 simulations of variable mass drug administration strategies. Because each strategy was implemented across a range (n = 3) of assumptions about the force of infection, a total of 18,522 simulations were created. The probability of achieving local interruption of transmission varied substantially across estimates of the force of infection and mass drug administration characteristics.

**Figure 2:**
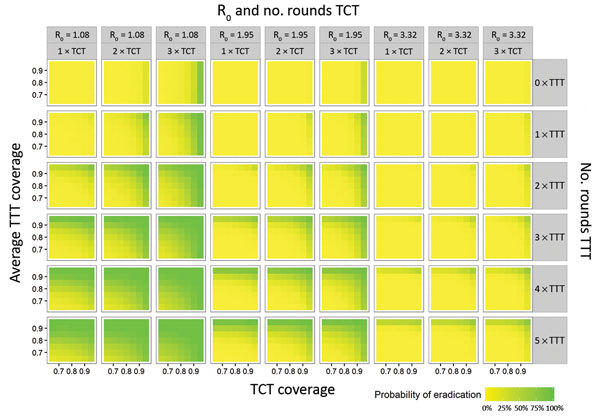
Predicted probability of achieving yaws eradication given variations in the estimate of R_0_ (basic reproduction number), total community treatment coverage, number of rounds of total community treatment, total targeted treatment coverage (TTT), and number of rounds of TTT. For this graph, we only show simulations where the coverage of persons with latent cases is the same as the coverage of persons with active cases during TTT. This might overrepresent the actual likelihood of achieving eradication because the coverage of persons with latent cases is probably lower than the coverage of persons with active cases during TTT. R_0_, basic reproduction number; TCT, total community treatment; TTT, total targeted treatment.

At the lowest estimates of the force of infection (R_0_ = 1.45) and with treatment rounds at 6-month intervals, the minimum treatment thresholds required to have a transmission interruption probability of >80% were a coverage of >75% of all populations and >8 rounds of treatment (3 rounds of TCT followed by 5 rounds of TTT). Increasing the coverage to 85% reduced the total number of rounds required to 5 (1–3 rounds of TCT followed by 2–4 rounds of TTT) (Figure 2; Table 2). For comparison, when the gap between treatment rounds was extended from 6 to 12 months, a total of 7 rounds of 85% coverage were required (2–3 rounds of TCT and 4–5 rounds of TTT).

**Table 2:** Indicative predicted coverage and number of rounds of treatment required to achieve yaws eradication

Predicted probability by estimated R_0_	Treatment every 6 mo			Treatment every 12 mo	
	Coverage required	Total no. rounds*		Coverage required	Total no. rounds*
80% predicted probability of eradication					
Low R_0_ (1.45, 95% CI 1.01–2.14)	75%	8		85%	7
Medium R_0_ (1.95, 95% CI 1.38–2.91)	90%	7		†	†
High R_0_ (2.47, 95% CI 1.7–3.68)	95%	8		†	†
100% predicted probability of eradication					
Low R_0_ (1.45, 95% CI 1.01–2.14)	85%	8		95%	6
Medium R_0_ (1.95, 95% CI 1.38–2.91)	†	†		†	†
High R_0_ (2.47, 95% CI 1.7–3.68)	†	†		†	†

*R_0_ (basic reproduction number) is the number of new cases arising from a single index case in a fully susceptible population. For this table, the number of rounds of total community treatment and total targeted treatment is combined (e.g., a total of 7 rounds could be 3 rounds of total community treatment and 4 rounds of total targeted treatment or, alternatively, 2 rounds of total community treatment and 5 rounds of total targeted treatment). Data are indicative only, and in some settings, higher coverage would allow a reduction in the total number of rounds required (see text and Figure 2).  †No combination of treatment variables was associated with the stated eradication probability.

At medium estimates of the force of infection (R_0_ = 1.95) and with treatment rounds at 6-month intervals , the equivalent thresholds were 90% coverage and a total of 7 rounds of treatment (2–3 rounds of TCT and 4–5 rounds of TTT) (Figure 2; Table 2). When the gap between treatment rounds was increased to 12 months, no combination of treatment variables was predicted to have a transmission interruption probability of >80%.

At the highest estimates of the force of infection (R_0_ = 3.32) and with treatment rounds at 6-month intervals, a total of 8 rounds (3 rounds of TCT and 5 rounds of TTT) with 95% coverage were required for a >80% likelihood of interrupting transmission (Figure 2; Table 2). When the gap between treatment rounds was increased to 12 months, no combination of treatment variables was predicted to have a probability of interrupting transmission of >80%.

We considered it plausible that, under field conditions, the coverage of persons with latent infection would not exceed 70% in any given round of TTT, because such cases are not clinically apparent, and adequate coverage might not be achieved by treating the immediate contacts of persons with clinical infection. At lower estimates of the force of infection, a total of 3 rounds of TCT with 85% coverage and 3 rounds of TTT (each with a coverage of persons with active infection of 85% and coverage of persons with latent infection of 65%) was associated with a >80% probability of interrupting transmission. If only 1 round of TCT was conducted, then coverage during TCT needed to be 90% and a total of 5 rounds of TTT (each with 90% coverage of persons with active infection and 65% coverage of persons with latent infection) were required. For medium estimates of the force of infection, a total of 8 rounds of treatment (3 rounds of TCT and 5 rounds of TTT) with a coverage of 90% were required. If only 1 round of TCT was undertaken, then 95% coverage was required, followed by 5 rounds of TTT with a 95% coverage of persons with active infection and 70% coverage of persons with latent infection. Under the highest estimate of the force of infection, no combination of treatment variables was associated with a high probability of interrupting transmission.

## Discussion

Our study demonstrates that, with implementation of the Morges strategy, interruption of transmission is possible in the setting considered. This finding suggests that eradication of yaws could be achieved, although caution must be applied because variability in the parameter estimates elsewhere could affect the effectiveness of these strategies. The parameter that has the strongest influence on whether elimination can be achieved is the transmission rate; that is, the rate at which infection occurs given contact between a susceptible and an infectious person (Technical Appendix Figure). We considered 3 different scenarios of the transmission rate of yaws based on serologic data, and our estimate of the feasibility of elimination varied considerably depending on these estimates. Further studies to obtain better estimates of the R_0_ in a range of countries where yaws is endemic would be of value to inform improved models and programmatic planning. 

A minimum of 8 rounds with coverage of >75% seems to be required for a high likelihood of achieving eradication but would prove inadequate at our highest estimates of possible values for R_0._ The predictions of our model are broadly in keeping with the real-world findings of the successful yaws elimination program in India (*9*), where 7 years of consecutive case finding and treatment (analogous to 14 rounds of TTT with 75% coverage) were conducted. In our model, the number of rounds of TTT also had a marked effect on the likelihood of achieving eradication, especially when coverage of persons with latent cases was limited to <70%. In these settings, the required number of rounds of treatment to interrupt transmission increased considerably.

Relatively few data are available on the transmission rate of yaws. Even within yaws-endemic countries, the prevalence of yaws varies markedly. Studies in the Pacific have found a seroprevalence of antitreponemal antibodies of >30% in several communities (*7*,*17*) and a prevalence of clinical yaws of ranging from 2.5% to 5% in communities before mass treatment. The prevalence of yaws is markedly lower in many yaws-endemic countries in West Africa (*20*), but limited community-based seroprevalence data are available to inform our understanding of disease transmission there. 

We modeled a range of estimates of R_0_ from 1.08 to 3.32 based on seroprevalence data and expert opinion. Given the substantial influence of these estimates on the likely outcome of community mass treatment, further studies to better understand disease transmission and how this varies within and between endemic communities would be of value. Ideally, these studies would obtain community-level, age-specific seroprevalence data that could be used to calculate the force of infection. No perfect serologic marker can be used for this task. Traditional treponemal serology combines a treponemal test, which reflects lifetime exposure but remains positive for life, with a nontreponemal test, the titer of which rises and falls after treatment. It is therefore not possible to use seroprevalence data to distinguish persons who have been infected many times from those who have been infected once, and seroprevalence estimates are likely to underestimate the actual force of infection. For this study, we calculated the force of infection while relying on treponemal serology alone, which should provide a more accurate estimate of the force of infection than if we used dual-positive serology. It remains, however, an imperfect measure.

Our model predicts that high coverage is required in all rounds of treatment to make yaws eradication feasible. Data from the previous WHO and United Nations Children’s Fund mass treatment campaign have highlighted the importance of achieving high coverage of persons with latent cases of yaws (*21*) and that treatment of persons with active cases alone is insufficient to interrupt transmission. These factors were important considerations in the adoption as part of the Morges strategy of an initial round of TCT regardless of the prevalence of active disease in a community. Given the high coverage requirement, particularly of persons with latent cases, and the relatively high fixed-costs of reaching yaws-endemic communities (*14*) compared with the relatively low costs of generic azithromycin, it might be preferable to conduct multiple rounds of TCT before the switch to TTT. Such a recommendation would be in line with the original Morges strategy (*22*), which recommended that additional rounds of TCT could be considered if the coverage achieved in the initial round of treatment was <90% or if access to yaws-endemic communities was difficult. A switch to multiple rounds of community mass treatment might also facilitate integration with other neglected tropical diseases mass drug administration programs in countries that are also frequently based on whole community mass treatment (*23*), although our model predicted a higher probability of achieving eradication with biannual treatment. Further studies to help determine the optimum strategy for achieving high coverage of persons with latent cases during the TTT phase of eradication efforts should be considered (e.g., studies of the spatial epidemiology of latent yaws cases in relation to persons with active cases in both pre– and post–mass drug administration settings or studies of whether additional mass treatment rounds specifically targeting children might be beneficial).

Our study has several limitations. Most notably, we lack accurate estimates for several disease parameters. The parameters used are derived from expert opinion and data from the Pacific region, and the transmission dynamics of yaws might be different in other regions of the world. However, the disease parameters used in this study are broadly in line with those used by other models of yaws transmission (*14*). We tested a range of coverage estimates for community mass treatment, but we did not factor in the possibility that some persons might be systematically missed during mass treatment campaigns, a phenomenon that has been observed in control efforts for other neglected tropical diseases (*24*). The current Morges strategy does not include adjunctive elements, such as water, sanitation, and hygiene interventions, in addition to mass treatment, although some studies suggest that improved access to water and sanitation is associated with a decreased risk for yaws (*17*). We did not include a secular trend in our model, and such a trend could be anticipated to further increase the likelihood of yaws eradication being achieved. Our model was designed to assess the feasibility of achieving yaws eradication in the near future, driven by the current WHO strategy, and in those conditions any effect of a secular trend could be expected to be minimal compared with the substantial impact of community mass treatment. Previous models have shown that secular trends are unlikely to substantially affect the cost-effectiveness of mass treatment (*14*); however, those models were based on an assumption of 90%–99% coverage in a single TCT round and 100% coverage of index patients and their contacts in the TTT round. More generally, we used a single-model structure that is simplified by modeling persons as being in 1 of a small number of disease-related compartments at any time and considering contact to occur at random. Uncertainty in model structure relating to disease progression and the probability of contact means that our findings should be interpreted carefully and potentially reassessed as elimination strategies are being applied.

In conclusion, our study assessed the theoretical achievability of worldwide yaws eradication and represents an important milestone in reaching the WHO’s eradication target. We have defined programmatic thresholds that might need to be met to achieve yaws eradication and identified key research questions to be addressed to inform refinements of the model and the worldwide roll-out of treatment strategies.

Technical Appendix Model design, implementation, and sensitivity analysis, and a graph of partial rank correlation coefficients of disease and treatment parameters with the probability of achieving yaws eradication.
