# Supplementary material for: Mathematical Modeling of Programmatic Requirements for Yaws Eradication
Source: Emerg Infect Dis. 2017 Jan;23(1):22–8. doi: 10.3201/eid2301.160487 (PMC5176210; doi:10.3201/eid2301.160487)
Supplement: Technical Appendix — Model design, implementation, and sensitivity analysis, and a graph of partial rank correlation coefficients of disease and treatment parameters with the probability of achieving yaws eradication. [file 16-0487-Techapp-s1.pdf]

Article DOI: http://dx.doi.org/10.3201/eid2301.160487

Mathematical Modeling of Programmatic Requirements for Yaws Eradication

Technical Appendix

Methods

Model Design

We developed a stochastic Markov model of community-level transmission of yaws (Figure 1). Upon infection, susceptible (S) individuals develop primary disease (I_1) at a rate proportional to the transmission rate β and the total number of infectious individuals ($I_1 + I_2$). Individuals with primary disease can further transition to secondary disease (I_2) at rate α, and both those with primary and secondary disease can transition to latent disease (L) at rate η. Lastly, those with latent disease can relapse back to I_2 at rate ρ. Infected individuals with primary or secondary disease are treated (and become susceptible again) at rate τ_I, and those with latent disease at rate τ_L. Unlike previous mathematical models of yaws (*1*), tertiary yaws was not included in the model as these cases are believed not to contribute to transmission (*2*). As individuals may be re-infected many times we did not consider individuals to obtain protective immunity following infection or treatment.

Implementing the Model

The Markov model was run with an optimized tau-leap algorithm to approximate Gillespie's algorithm for exactly simulating stochastic systems (*3*,*4*).

We used the *adaptivetau* package for R (the R Project for Statistical Computing) (*5*). We performed repeated simulations across a range of disease transmission assumptions (β, n = 3) and treatment assumptions, varying both the coverage and number of mass treatment rounds undertaken (Table 1).

For each combination of disease and intervention parameters we performed 1,000 simulation experiments. Within each combination we used Latin-hypercube sampling using the *lhs* R package (*6*) to vary other disease specific variables (rate of progression and relapse,

treatment in the absence of intervention) across the range of parameter estimates. We included an initial burn-in time of 50 months before the commencement of any yaws eradication intervention. The model then ran for a further 100 months.

Sensitivity Analysis

Initial sensitivity analysis was conducted by varying both disease (β) and intervention (coverage, number of rounds of MDA) and analyzing alterations in the eradication probability. In addition, we calculated Partial-Rank Correlation Coefficients for all model parameters (disease and treatment), which calculates the correlation of each variable with the probability that eradication is achieved if all other parameters are kept fixed, as previously described (*7*).

Code for the Model

Available at https://github.com/michaeledwardmarks/yaws-model-code.

References

1. Fitzpatrick C, Asiedu K, Jannin J. Where the road ends, yaws begins? The cost-effectiveness of eradication versus more roads. PLoS Negl Trop Dis. 2014;8:e3165. PubMed http://dx.doi.org/10.1371/journal.pntd.0003165

2. Marks M, Mitjà O, Solomon AW, Asiedu KB, Mabey DC. Yaws. Br Med Bull. 2015;113:91–100. PubMed http://dx.doi.org/10.1093/bmb/ldu037

3. Cao Y, Gillespie DT, Petzold LR. Efficient step size selection for the tau-leaping simulation method. J Chem Phys. 2006;124:044109. PubMed http://dx.doi.org/10.1063/1.2159468

4. Gillespie DT. Exact stochastic simulation of coupled chemical reactions. J Phys Chem. 1977;81:2340–61. http://dx.doi.org/10.1021/j100540a008

5. Johnson P. Adaptivetau: Tau-leaping stochastic simulation. R package version 2.2 [cited 2016 Mar 21]. https://CRAN.R-project.org/package=adaptivetau

6. Carnell R. LHS: Latin hypercube samples. R package version 0.13 [cited 2016 Mar 21]. https://CRAN.R-project.org/package=lhs.

7. Wu J, Dhingra R, Gambhir M, Remais JV. Sensitivity analysis of infectious disease models: methods, advances and their application. J R Soc Interface. 2013;10:20121018. PubMed http://dx.doi.org/10.1098/rsif.2012.1018

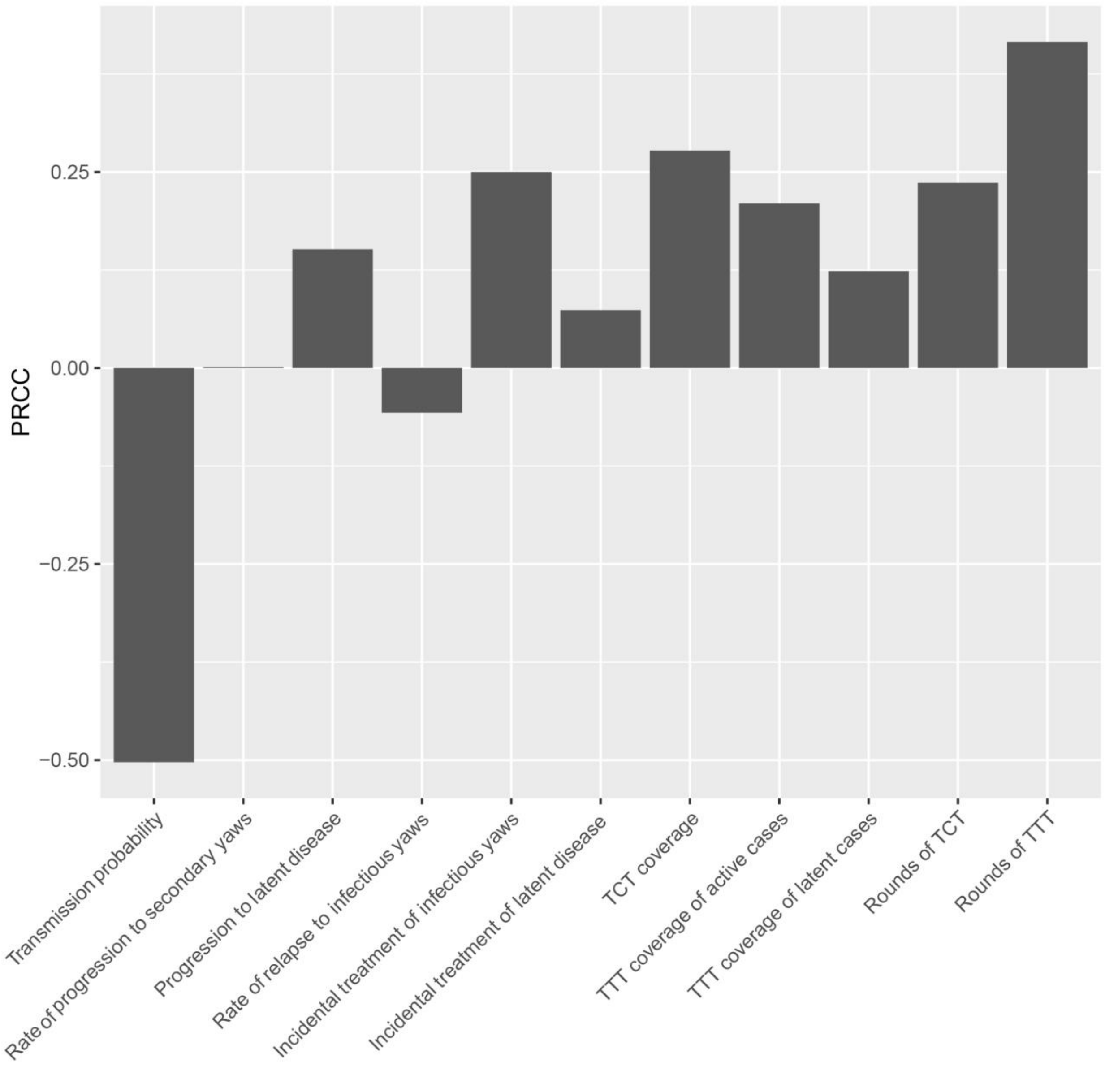

Technical Appendix Figure. Partial rank correlation coefficients of disease and treatment parameters with the probability of achieving yaws eradication. PRCC, partial rank correlation coefficient; TCT, total community treatment; TTT, total targeted treatment.
